# Learning and Action in Community Health: Using the Health Belief Model to Assess and Educate African American Community Residents about Participation in Clinical Research

**DOI:** 10.3390/ijerph15091862

**Published:** 2018-08-28

**Authors:** Latrice Rollins, Angela Sy, Nicole Crowell, Desiree Rivers, Assia Miller, Pamela Cooper, Debra Teague, Cassandra Jackson, Tabia Henry Akintobi, Elizabeth Ofili

**Affiliations:** 1 Department of Community Health and Preventive Medicine, Morehouse School of Medicine, 720 Westview Dr., Atlanta, GA 30310, USA; drivers@msm.edu (D.R.); amiller@msm.edu (A.M.); takintobi@msm.edu (T.H.A.); 2 John A. Burns School of Medicine, University of Hawai’i at Manoa, 651 Ilalo Street, BSB 320, Honolulu, HI 96813, USA; sya@hawaii.edu; 3 Clinical Research Center, Morehouse School of Medicine, 720 Westview Dr., Atlanta, GA 30310, USA; ncrowell@msm.edu (N.C.); pcooper@msm.edu (P.C.); dteague@msm.edu (D.T.); cjackson@msm.edu (C.J.); eofili@msm.edu (E.O.)

**Keywords:** biorepository, clinical research, minority participation, African Americans, community engagement, health belief model

## Abstract

The Learning and Action in Community Health project was implemented to gather preliminary data needed to inform community-engaged educational approaches to increase clinical research participation among racial minorities. The Health Belief Model was the theoretical framework utilized to develop the intervention and assessment tools. An educational session about clinical research and biorepository participation was designed using clinicaltrials.gov information and administered to adult, African American community residents (*n* = 60) in Atlanta, Georgia. Pre- and post-tests were collected and analyzed to assess changes in participants’ knowledge, perceptions, and willingness to participate in clinical studies and biorepositories. There were statistically significant changes in knowledge about joining a clinical study (*p* < 0.001) and registry or biorepository (*p* < 0.001). There was no statistically significant change in willingness to participate in clinical research or biorepositories after the educational session. Focus groups were conducted to gather feedback on the educational session and perceived barriers and benefits to participating in clinical research. Perceived benefits were improving health, receiving incentives, early detection of health issues, and access to care. Perceived barriers included fear, lack of knowledge, historical mistrust of research, and time constraints. Results have implications for subsequent community-engaged approaches to increasing minority participation in clinical research.

## 1. Introduction

Low engagement of racial minorities in health research is well recognized in biomedical, clinical and translational research [1,2,3]. Despite decades of initiatives to include participation of minorities in research, participation of adult minorities in clinical trials (CT) is not proportional to their representation in the U.S. population [4,5]. Racial minorities still have lower participation rates in medical research, which includes biospecimen collection for research purposes. Only 5.25% of cases in the Cancer Genome Atlas, a National Cancer/National Human Genome Research Institute- developed and supported biobanking program, were collected from African Americans [6]. In cancer CTs, African Americans represented less than 10% of research participants enrolled [7]. Finally while 62% of adults participating in HIV medication trials were White, only 23% were Black [8].

Consequently, for more than a decade, investigators have published on the important issue of increasing racial minority representation in research [9,10,11]. The low rate of CT enrollment of African Americans has been attributed to the lack of information regarding clinical trials and participation opportunities, individual-characteristics (e.g., age, socioeconomic status, and comorbid conditions), and concerns about CTs (e.g., trial setting, dislike of randomization for study participation, potential side-effects) [12,13]. A systematic review of the factors influencing African Americans’ participation in cancer CTs found five important elements related to CT participation: (1) negative beliefs; (2) lack of knowledge; (3) influence of faith; (4) health care providers’ role; and (5) friends’ or relatives’ previous participation recommendation [14]. 

Current studies on CTs in general, cancer CTs specifically, medical research participation, and biospecimen research donation have all indicated that knowledge of past research abuses are no longer the major factor influencing present decisions to participate in CTs [15]. These studies found that demographic factors such as age and level of education were related to identifying past research abuses as a barrier to CT and medical research participation [7,16,17]. The studies on barriers against cancer CT participation and biospecimen research donation both found that past research abuses are not a major factor in participation, including the finding that participants in fact knew very little, if anything at all, about past research abuses [15,17]. 

More recently, participation of racial minorities in CTs has been low because of limited awareness about participating in research itself. Relative to the White population, racial minority participants have been found to be persistently less aware of CTs and less positive about the use of medical information for research [16,18]. Furthermore the most common reasons cited by African Americans in a study on biospecimen research donation cited lack of knowledge of how biospecimens will be used and about biospecimens in general [17]. When patients were educated about cancer CTs and given relevant reasons to participate, awareness and acceptance of research participation were found. Furthermore tailored education brought to communities through outreach can improve attitudes and acceptance of clinical research [19,20]. 

The field of health disparities research lacks scientific consensus about how best to respectfully recruit underrepresented minority populations in research [5]. According to the National Academies of Sciences (2016), the lack of participation of racial minorities in research is because most (White) researchers provide less time, attention and resources required to effectively engage minority populations in research [3]. When recruiting vulnerable communities in research, researchers should ensure that these potential participants fully understand the research, their potential risks, and the implications of their participation (i.e., research literacy). 

### The Learning and Action in Community Health Project 

In 2007, the Research Centers in Minority Institutions (RCMI) was established promoting inter-institutional collaborations among 18 institutions to better understand and address diseases etiology and treatment within underserved and vulnerable populations. The RCMI Translational Research Network (RTRN) is a National Institutes of Health-funded project to leverage expertise and resources in research collaborations from across RCMI institutions. RTRN fosters and supports inter-institutional collaboration to leverage outcomes, resources and expertise across RCMI institutions. RTRN aims to enhance collaborative research capacity toward the understanding and treatment of diseases, with a focus on racial minorities. One of the aims of the RTRN is to translate research knowledge gained to and from communities in culturally- and linguistically-appropriate and cost-effective ways that reflect our evolving demographics. 

The translation of basic science research discoveries into evidence-based medicine and public health practice requires inter-institutional and multi-disciplinary collaborations. RTRN is comprised of the Administrative Coordinating Center (ACC), Research Coordinating Center (RCC), and Data Coordinating Center (DCC) with experienced and proficient principal directors, clinicians, data managers, statisticians, and communications and community professionals. The Coordinating Centers work collaboratively to support the investigators from the RCMIs in their collaborative, multi-site research with Network partners [21].

As part of the RTRN RCC, its Community Engagement Core aims to foster collaboration and disseminate accurate and easy to understand information about health research. End users include academic investigators, community organizations, community researchers, and industry researchers. Anticipated outcomes include but are not limited to translational research that yields health-promotion and disease-prevention interventions and clinical care that is relevant to and accessible by community members and feasible for implementation by programs that serve them. 

Investigators from the RTRN ACC, which includes an Evaluation Core, and Community Engagement Cores developed and implemented a key initiative, called Learning and Action in Community Health (LACH), that would benefit the RCMI communities, identifying barriers and facilitators to clinical research participation, to further develop information and resources for RTRN communities and researchers. The initiative was established as a pilot, community-engaged intervention, launched at Morehouse School of Medicine among African American adults in Atlanta, GA. The primary aim of LACH was to increase African Americans’ knowledge about clinical research and willingness to participate in clinical research and improve their perceptions and attitudes about clinical research. This paper describes the design and results of the LACH project.

## 2. Materials and Methods 

### 2.1. Theoretical Framework

**Figure 1:**
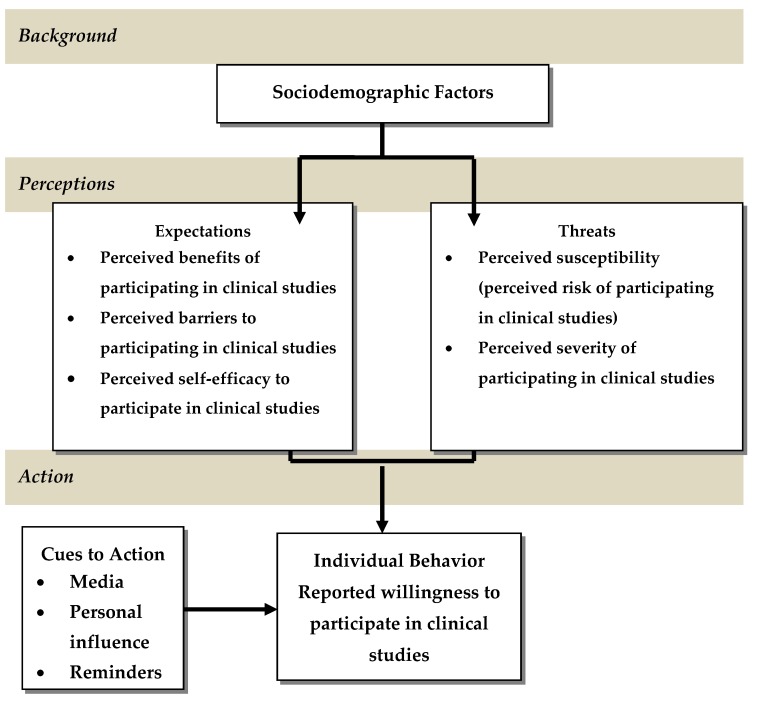
Health Belief Model.

A conceptual framework based on the Health Belief Model (HBM) was developed to guide this study. The HBM has been used in previous studies to identify factors that can affect the degree of success in recruiting and retaining ethnically diverse populations in research. The HBM was developed during the early 1950s by a group of U.S. Public Health Service social psychologists and was one of the first theories of health behavior [22]. Initially, psychologists applied the HBM to understand and explain the low rates of participation of the public in various disease prevention and detection programs [22]. Later, the model was extended to study people’s responses to symptoms and their behaviors in response to a diagnosed illness, particularly adherence to medical regimens [22,23,24]. The HBM correlates factors such as attitudes, beliefs, and perceptions about a particular health condition or health behavior with the actual practice of that behavior. The fundamental principle of the model is that an individual’s perceived risk of being affected by a particular health condition and the perceived severity of these effects impacts decision-making behaviors [22]. The HBM is composed of six constructs which are believed to influence people’s decisions to take action to prevent, screen for, and control illness [22]. The first construct is perceived susceptibility. Perceived susceptibility is defined as the perceived threat of illness, consisting of the level of personal susceptibility to the particular illness or condition [25]. The second construct, perceived severity, refers to a person’s belief about the degree of severity of the negative consequences (organic, social or both) which might result from contracting the condition [25]. The third construct is perceived benefits. Perceived benefits are a person’s belief that their health behavior will be beneficial or effective in preventing or reducing susceptibility or severity of disease [25]. The fourth construct is perceived barriers. Perceived barriers are defined as barriers or costs that are physical, psychological, and financial against initiating or continuing the advocated behavior [25]. Cue to action is the fifth construct, which is a stimulus to trigger the desired health behavior where the person is consciously aware of his feelings about the health threat. These cues are internal (perception of painful or uncomfortable physical sensation) or external (mass media, interpersonal interactions) [25]. The final construct is self-efficacy, the beliefs about one’s personal ability to perform behaviors that bring desired outcomes [25]. Self-efficacy is also referred to as personal control, the perception that one has the ability, resources, or opportunities to obtain positive outcomes or avoid negative effects through one’s own actions [26]. Personal control is an important predictor of health behaviors. According to Seligman (1975), research on animals and humans has found that feelings of helplessness generally decrease attempts to change one’s situation even when effective action is available [26]. Figure 1 illustrates how the HBM is applied in this study.

Risk perception and how an individual perceives the possible threat of a disease or health behavior on their life can have significant impact on informed decision-making and health behavior practices. Accurate risk perception is important because it is imperative to informed decision making among patients [27]. 

### 2.2. Study Design

The study employed a mixed methods design with qualitative and quantitative components through administration of 19-item pre- and post-surveys and focus groups to a purposive sample of African American adults. The study protocol, design and intervention were developed, reviewed and approved by the investigators from the RTRN ACC and Community Engagement Cores. This study was reviewed by MSM’s Institutional Review Board in May 2016 (project identification code 910493-4). 

### 2.3. Study Population

Inclusion criteria for the study were African American adults, 18 years old and older residing in Atlanta, GA. A total of 60 African American adults were recruited in June 2016. The Morehouse School of Medicine (MSM) RCMI Clinical Center’s Recruitment and Retention Core and the Community Physicians’ Network supported the LACH Project by providing strategies and resources based upon the target demographics and number of participants needed to meet the targeted enrollment for both the surveys and focus groups. There were preliminary discussions toward developing a strategic plan to engage various sources identified as external or internal community engagement partners and the MSM RCMI Clinical Center’s Community Advisory Board. The strategic plan involved utilizing sources such as social media, word of mouth and information from other projects where participants agreed to be contacted for future studies. Individuals who completed the 1-hour educational session, both the pre- and post-session surveys, and participated in a focus group were compensated with a $50 Walmart gift card. 

### 2.4. Intervention

The intervention consisted of a 1-hour educational session conducted at MSM. The session was offered on two days in the evening and participants could choose which day they would like to attend. The session entitled, What You Need to Know about Clinical Studies, was facilitated by the RTRN program manager. The content was based on information found on clinicaltrials.gov and the National Cancer Institute. The session topics included: definitions of biospecimens, biorepositories, patient registries, clinical studies, clinical trials; what happens during a clinical trial (e.g., phases of clinical trials); observational studies; who conducts clinical studies; where clinical studies are conducted; how long clinical studies last; reasons for conducting clinical studies; how to participate in a clinical study; how participants are protected; the role of institutional review boards; the relationship between clinical studies and regular healthcare; and questions participants should ask before participating in a clinical study. 

### 2.5. Quantitative Data Collection

Pre- and post-session surveys were administered to LACH participants in educational sessions. The 19-item surveys included questions that were developed to evaluate LACH participants’ perceptions of constructs in the HBM. Specifically, the pre- and post-surveys were distributed to gather information on changes in knowledge, perceptions and willingness to participate in clinical studies and biorepositories before and after the educational session. Table 1 shows how each HBM construct was operationally applied to the study. 

### 2.6. Quantitative Data Analysis

Pre- and post-surveys were analyzed, comparing frequencies and means for each outcome variable from pre- to post-test and between pre- and post-intervention groups. The responses were ranked using a Likert type 5-point scale ranging from 1 to 5 (Strongly Disagree to Strongly Agree). We used Fisher Exact test and Wilcoxon–Mann–Whitney tests to compare the groups, with a *p*-value of <0.05 to differentiate the statistical significance of outcomes between study groups. If the parameter was statistically significant, then we concluded that there was the difference in outcome between groups. We reported means, standard deviations, and *p*-values. Statistical analyses were carried out using SAS version 9.2 (SAS Institute Inc., Cary, NC, USA).

### 2.7. Qualitative Data Collection

After completing the post-educational session survey, participants were split into two groups to participate in a focus group. Four focus groups were facilitated by RTRN evaluation staff in June 2016. All 60 participants participated in a focus group. Focus groups lasted 60–90 min and were guided by a standardized discussion guide. Focus group questions included perceptions associated with participation in clinical research or biorepositories (i.e., perceived barriers, benefits), and cues to action (i.e., how to share information, community partnership suggestions). 

### 2.8. Qualitative Data Analysis

The focus groups were audio-recorded and transcribed verbatim for content and data analysis. Additionally, hand-written notes were taken by the co-moderator to supplement the transcripts with behavioral observations made during each focus group. Results from the focus group transcripts were analyzed initially using a study codebook with a priori codes, derived from the HBM framework. The study codebook expanded with emergent codes, identified as data analysis progressed. In accordance with best practices and previous research, two members of the study team coded each transcript independently, and coded transcripts were compared to ensure intercoder agreement [28,29]. Data were analyzed using a combination of content analysis and the constant comparison method [30,31,32]. The HBM framework was used to group narrow themes more broadly to form overarching themes of perceived expectations, perceived threats, and factors that influenced action (i.e., participation). Thematic saturation was assessed and achieved.

## 3. Results

### 3.1. Quantitative Results

A total of 58 participants provided their demographic information in survey completion. Table 2 provides detailed information on the characteristics of participants based on pre-test surveys. Almost half of participants, 46.66% were in the 40–60-year-old age group. All respondents classified themselves as African American and 53.33% identified themselves as female. Almost one quarter (24.13%) had some or completed high school, and the majority (75.87%) had at least one year of college or higher education completion. Most (96.67%) respondents noted that English was the language most frequently written and spoken (95%).

A total of 60 participants completed pre-and post-session surveys. Table 3 displays results of the pre- and post-intervention assessment and includes two items to identify the effect of the educational session on willingness to take part in a clinical research study and share biological samples to a registry or biorepository. A few answers to the two following questions showed a marginal increase from pre- to post-test “Are you willing to take part in a clinical research study?” (*p* = 0.8850) and “Are you willing to give your biological samples to a registry or biorepository?” (*p* = 0.0011) that are not statistically significant.

Information on knowledge, perceptions and willingness to participate in clinical studies and biorepositories before and after the educational session were also assessed and several items showed a marked change from pre- to post-session survey (Table 4). There was a statistically significant increase in agreement with statements “I know how to join a registry or biorepository.” (*p* < 0.0001) and “I know how to join a clinical research study.” (*p* < 0.0001). Though a few other items showed increases in numbers of affirmative statements, they did not reach statistical significance. 

### 3.2. Qualitative Results: Descriptive Analysis of Focus Group Data

#### 3.2.1. Perceived Benefits of Clinical Research Participation

Focus group participants were asked “What do you think are the benefits of participating in clinical research or participant registries?” Common themes that the participants reported were: helping to improve health, receiving incentives, early detection, and access to healthcare/medications. Participants’ responses indicated that a perceived benefit of clinical research participation was helping to improve health:

*I am not a proponent of drug use as the treatment of choice, but some drugs do help some people in certain situations and it’s absolutely essential that those drugs be tested before they’re released to the public. So it has its place. I believe it is important*.

Some participants mentioned helping others but primarily expressed that receiving compensation was a perceived benefit:

*Each generation, each age, each color, there’s the benefits
of knowing that it can help somebody else out. But the main compensation coming
from the hood would be getting paid*.

Finally, some participants noted that because of lack of health insurance and access to health care, participating in clinical research could be a means of early detection of health issues and access to healthcare or medications. One participant stated:

*With these studies that are prevalent now and targeted for
minority environments, the way I see it is that {it} affords them an
opportunity to get medical attention that perhaps they couldn’t have gotten
earlier because they did not have proper medical attention*.

#### 3.2.2. Perceived Barriers to Clinical Research Participation

Focus group participants were asked “What do you think would discourage someone from participating in clinical research or participant registries?” Common themes that participants shared were: Fear, lack of knowledge, history (Tuskegee), and time. Participants’ responses indicated that the top threats to participation were fear and lack of knowledge:

*Prior to this experience I would never have considered
participating because I would feel like a guinea pig. Like they’re wanting me
to sample something that they don’t even know what the effects are. So I
wouldn’t have considered it because I was afraid*.

Several participants also noted history, specifically the Tuskegee study, still having lasting negative impact on African Americans’ perceptions of clinical research:

*I think we have a checkered past here in the United States. They gave Indians smallpox. They gave black guys syphilis and then refused to give them the treatment. So a lot of people in our community think about things like that before they will go somewhere and get stuck or give blood*.

Finally, participants also noted not taking the time to participate in clinical research as a barrier. One millenial noted:

*I think too is just where like my age group just don’t want
to take the time to really participate*.

#### 3.2.3. Cues to Action 

Focus group participants were asked about different ways that participation in clinical research could be encouraged in African American communities. Participants were asked “What are the most appropriate methods for education about clinical research and participant registries for minority community members?” The most common themes that participants mentioned were: the use of layman’s language, social media, word of mouth, health and job fairs, and social and educational intervention format. Participants suggested that one way to encourage participation was ensuring layman’s language is used in all communication,

*Many proposals for the various studies are written from the academic perspective, and meeting their standards. However, I think it was at a sixth or eighth grade level that every proposal should be—somebody in that region should be able to see it, read it, and say okay this is what I’m going to be a part of*.

Several participants thought the use of social media would be effective:

*So to me social media, I’m not going to say is the media, but it is a reliable platform for me. If you think past just a quick selfie that people are posting or just a quick little videos that are being passed on every day, it can be helpful and beneficial to engage people directly like in groups or have a video that’s shared from a group or something like that. So it can be a useful platform*.

Many participants indicated that word of mouth or hearing from a trusted friend or family member would encourage participation:

*So if a black person knows that their auntie is going a lot and she’s getting all of these benefits or perks or whatever you want to call them, then maybe she’ll go, she’ll tell her friend, and that cycle will continue*.

Additionally, participants also noted that information about clinical research could be shared at health and job fairs.

*And you have health fairs. A lot of people don’t have
insurance even with the Obamacare and I know a friend of mine,* *she goes to
health fairs to get her mammogram and blood work and everything because she
doesn’t have the money. So a lot of times health fairs, that’s what people are
going for now—for health fairs because they’re offering to check your diabetes,
check this, check that. When you don’t have the funds and you want to stay as
healthy as you possibly can but sometimes your pocketbook doesn’t let you. You
know what I mean?*

*A few years ago I went to the job fair hosted by the City of
Atlanta. And out of the 80 vendors that were there, 20 of them were actually
selling products. What would be great is if people had this knowledge {of
clinical research opportunities}*.

Finally, the participants thought that using a “social” or community-engaged approach that was utilized by LACH to provide education and receive feedback was effective.

*Culturally we’re very social so that’s certainly a good place
to start. I think any time you make something lecture style you’re going to
lose quite a bit of people. It’s just—that’s just how it is*.

Focus group participants were also asked, “What organizations (national, state, local) or businesses should clinicians or researchers work with in sharing clinical research or participant registry information in your community?” The most frequently mentioned potential community partners were churches, community groups, local policymakers, schools, Black Greek organizations, and professional organizations, such as American Association of Retired Persons (AARP):

*Because a lot of us attend church and we listen to our
pastors and church leaders*. *We trust them*

*I’m 18, so …we could also go to schools … and they would have
people … come to the school and talk to them about it*.

*Maybe presidents of our homeowners associations or like the
neighborhood watch leaders, Boys and Girls Club presidents, things like that*.

## 4. Discussion

This study provides new insights regarding willingness of African American community residents to participate in clinical research. LACH was designed to increase African Americans’ knowledge about clinical research and willingness to participate in clinical research and improve their perceptions and attitudes about clinical research. Knowledge, perceptions and willingness to participate in clinical studies and biorepositories before and after an educational session were assessed. The results suggest that the educational session increased participants’ self-efficacy regarding their ability to take part in a clinical study as demonstrated by the statistically significant increase in participants’ agreement with statements, “I know how to join a registry or biorepository.” (*p* < 0.0001) and “I know how to join a clinical research study.” (*p* < 0.0001). 

However, LACH participants were not more willing to participate in clinical studies as demonstrated by participants’ responses to questions, “Are you willing to take part in a clinical research study?” (*p* = 0.8850) and “Are you willing to give your biological samples to a registry or biorepository?” (*p* = 0.0011). It is possible that there was no statistically significant change in willingness to participate in clinical research or biorepositories after the educational session, because the majority responded they were willing to participate during the pre-test or before the intervention. Also there was statistically insignificant decrease in the number of participants who reported that they are willing to take part in a clinical research study. It could be explained that few participants answered “Maybe” instead of “Yes”. 

Focus group results describe reasons related to participating and not participating in clinical research and were related to knowledge and self-efficacy. To address the barriers of distrust and fear, participants recommended that having knowledge and learning about research participation would dispel such attitudes. Participants provided specific suggestions on ways to provide education and knowledge to the community such as sharing information in the church, formal school settings, or through contemporary channels as social media. Using social media is a way for friends and relatives to communicate with each other about research participation while churches are a trusted source of also providing education and knowledge.

Study results are both consistent with previous finding while adding new information. LACH focus group findings support the existing literature on barriers to African American participation in clinical research, including the lack of information or knowledge about clinical research [13]. Further, this study supports existing research that facilitators of African Americans’ participation in clinical research is friends’ or relatives’ previous participation in clinical research or friends’ or relatives’ recommendation (i.e., word of mouth) [15]. Despite research indicating that distrust of the medical system and of research by racial minorities and knowledge of past research abuses are no longer the major factor influencing present decisions to participate in clinical trials, LACH participants frequently cited fear and the history of unethical research as barriers to participation [12,15,33].

The study also had some limitations. First, the result from African American participants in Atlanta may have limitations in generalizing to African Americans in other communities or to other racial minorities. Second, the effectiveness of the educational session was evaluated by surveys with multiple-choice questions. Although the pre- post-session surveys were developed based on a theoretical framework, using a validated instrument to confirm our findings in further studies is needed. Finally, recent research suggested that willingness to participate does not reflect actual participation as participants may be more likely to answer positively about willingness to participate due to a tendency to exhibit pleasing and socially accepted behavior [34].

## 5. Conclusions

The results of this pilot initiative to increase African American knowledge and willingness to participate in clinical research are promising and offer suggestions for tailoring the educational content. This study demonstrates that the HBM is a useful model for understanding and addressing the often complex reasons why racial minorities’ have perceived barriers and benefits of participating in clinical research. This study also indicates the strength of mixed methods, community-engaged assessments of African Americans’ perceptions of clinical research. The focus group sessions provided context, regarding perceived benefits, barriers, and best approaches to engage respective demographic groups.

Future implementation of the LACH project could train enrolled participants to encourage others to partake in clinical research and become community champions or citizen scientists, to educate and promote the importance of clinical trials and focus group participation, using the suggested channels and partners (e.g., churches, social media). This study thereby builds on the evidence and provides timely recommendations critical to educating and engaging racial minorities in clinical research. 

## Figures and Tables

**Table 1:** Health Belief Model: Constructs examined and defined in this study.

Constructs	Operationally Defined in this Study by the Corresponding Survey Question(s)
Cues to action leading to individual behavior	Willingness to take part in a clinical research study. Willingness to give biological sample to a registry or biorepository.
Perceived susceptibility	I need to agree before joining a clinical research study. I need to agree before joining a research registry or biorepository. My personal information is protected when I join a research study.
Perceived benefits	Joining a clinical research study will help me get new health treatments that the public can’t get yet. Joining a clinical research study may help me get better medical care. Joining a clinical research study gives me more control over the health care I get. Joining a clinical research study allows me to help others by giving to medical research.
Perceived barriers	Taking part in a clinical research study will take too much of my time. I’m able to get transportation to take part in a clinical research study. My health insurance will not pay for me to take part in a clinical research study. People who oversee clinical research studies may not tell me all the risks of taking part in the study.
Perceived severity	The risks of taking part in a clinical research study are more than the benefits. There may be some serious side effects from taking part in a clinical research study. Taking part in a clinical research study could put my life at risk. When I think about joining a clinical research study it scares me.
Self-efficacy	I know how to join a biorepository or registry. I know how to join a clinical research study. I can talk to my doctor about joining a research study.

**Table 2:** Baseline characteristics between study groups (*n* = 58).

Characteristics	Number	Percent
Age		
18–30	12	20.00
31–40	9	15.00
41–50	14	23.33
51–60	14	23.33
61+	11	18.33
Sex		
Male	28	46.67
Female	32	53.33
Race/ethnicity		
Black or African American	58	100.00
Highest grade or year of school completed		
Grades 9 through 11 (Some high school)	1	1.72
Grade 12 or GED (High school graduate)	13	22.41
College 1 year to 3 years (some college or technical school)	24	41.38
College 4 years (College graduate)	11	18.97
Graduate school (Advanced degree)	9	15.52
Language most frequently written		
English	58	96.67
Language most frequently spoken		
English	57	95.00

**Table 3:** Effects of educational session: Results of Fisher Exact Test (*n* = 60).

Survey Items		Pre-Test N (%)	Post-Test N (%)	Fisher Exact Test *p* Value
Are you willing to take part in a clinical research study?	Yes	47 (51.6)	44 (48.4)	0.8850
	No	3 (50.0)	3 (50.0)	
	Maybe	10 (45.5)	12 (54.5)	
Are you willing to give your biological samples to a registry or biorepository?	Yes	36 (48.0)	39 (52.0)	0.4771
	No	3 (37.5)	5 (62.5)	
	Maybe	21 (58.3)	15 (41.7)	

**Table 4:** Knowledge, perceptions and willingness to participate in clinical studies and biorepositories: Results of Wilcoxon–Mann–Whitney test.

Survey Items	Pre-Test	Post-Test	Wilcoxon-Mann-Whitney Test
	Mean (SD)	Mean (SD)	*p* Value
I need to agree before joining a clinical research study.	4.53 (0.87)	4.59 (0.75)	0.8472
I need to agree before joining a research registry or biorepository.	4.55 (0.78)	4.65 (0.66)	0.5486
I know how to join a registry or biorepository.	2.69 (1.17)	3.83 (1.04)	<0.0001 *
I know how to join a clinical research study.	3.10 (1.19)	4.00 (1.00)	<0.0001 *
The risks of taking part in a clinical research study are more than the benefits.	2.84 (1.13)	2.76 (1.03)	0.5839
Joining a clinical research study will help me get new health treatments that the public can’t get yet.	3.35 (1.00)	3.53 (0.83)	0.2991
Joining a clinical research study may help me get better medical care.	3.48 (0.83)	3.40 (0.98)	0.8103
Joining a clinical research study gives me more control over the health care I get.	3.12 (0.97)	3.25 (0.96)	0.4592
Joining a clinical research study allows me to help others by giving to medical research.	4.13 (0.89)	4.10 (0.93)	0.8575
There may be serious side effects from taking part in a clinical research study.	3.27 (1.07)	3.34 (1.12)	0.6069
Taking part in a clinical research study could put my life at risk.	2.75 (1.06)	2.91 (1.10)	0.4446
Treatment given to me for a clinical research study may not help me.	3.21 (1.08)	3.51 (1.08)	0.1115
Taking part in a clinical research study will take too much of my time.	2.41 (1.02)	2.25 (0.89)	0.4946
When I think about joining a clinical research study it scares me.	2.16 (1.02)	2.25 (1.08)	0.6178
I’m able to get transportation to take part in a clinical research study.	4.03 (0.99)	3.93 (1.10)	0.7090
My health insurance will not pay for me to take part in a clinical research study.	3.51 (1.02)	3.48 (0.95)	0.8599
People who oversee clinical research studies may not tell me all the risks of taking part in the study.	2.62 (1.12)	2.37 (1.07)	0.2471
My personal information is protected when I join a research study.	3.81 (1.04)	4.08 (0.87)	0.1684
I can talk to my doctor about joining a research study.	3.92 (1.00)	4.18 (0.85)	0.1307

* Significant at *p*-value < 0.05.
